# *Trichoderma* Enzymes for Degradation of Aflatoxin B1 and Ochratoxin A

**DOI:** 10.3390/molecules27123959

**Published:** 2022-06-20

**Authors:** Irene Dini, Vittoria Alborino, Stefania Lanzuise, Nadia Lombardi, Roberta Marra, Anna Balestrieri, Alberto Ritieni, Sheridan L. Woo, Francesco Vinale

**Affiliations:** 1 Department of Pharmacy, University of Naples Federico II, Via Domenico Montesano 49, 80131 Naples, Italy; alberto.ritieni@unina.it (A.R.); sheridanlois.woo@unina.it (S.L.W.); 2 Department of Agricultural Sciences, University of Naples Federico II, Via Università 100, 80055 Portici, Italy; agr.vittoria.alborino@gmail.com (V.A.); stefania.lanzuise@unina.it (S.L.); nadia.lombardi@unina.it (N.L.); robmarra@unina.it (R.M.); 3 BAT Center—Interuniversity Center for Studies on Bioinspired Agro-Environmental Technology, University of Naples Federico II, Via Università 100, 80055 Portici, Italy; 4 Department of Animal Health, Istituto Zooprofilattico Sperimentale del Mezzogiorno, Via della Salute 2, 80055 Portici, Italy; anna.balestrieri@izsmportici.it; 5 Department of Veterinary Medicine and Animal Productions, University of Naples Federico II, Via Federico Delpino 1, 80138 Naples, Italy

**Keywords:** *Trichoderma*, culture filtrate, Aflatoxin B1, Ochratoxin A, mycotoxin, peroxidase activity, exoenzymes

## Abstract

The contamination of agricultural products with mycotoxins causes risks to animal and human health and severe economic losses. Mycotoxicoses can be reduced by preventing fungal infection using chemical and biological approaches. The chemical strategies can release toxic molecules; therefore, strategies for biological control are being evaluated, such as using nontoxic fungi and their metabolites. This work evaluated the effect of exoenzymes produced by the beneficial fungus *Trichoderma afroharzianum* strain T22 in degrading Aflatoxin B1 (AFB1) and Ochratoxin A (OTA). The ability of *Trichoderma* to produce hydrolases was stimulated by using different inducing substrates. The highest AFB1 and OTA degradation activity was obtained using a medium containing lyophilized mushrooms and crude fiber. The *T. afroharzianum* T22’s ability to reduce mycotoxins may be attributed to peroxidase enzymes. This study showed that *T.* *afroharzianum* strain T22 or its peroxidase supplementation could represent a sustainable strategy for the degradation of AFB1 and OTA in feed and food products.

## 1. Introduction

Mycotoxins are secondary metabolites produced by plant-parasitic fungi, which can produce acute or chronic diseases in animals and humans [1]. Some of these compounds are lipophilic molecules mainly absorbed in the intestine and distributed in fat and soft tissues [2], with subsequent detoxification and detriment of the kidney and liver [3]. The International Agency for Research on Cancer (IARC) classified some mycotoxins (i.e., Aflatoxin B1-AFB1 and Ochratoxin A-OTA) as the most potent natural carcinogens [4]. Mycotoxins contamination can occur in the field or during processing and storage periods. Cereals; fruits; coffee; cocoa; herbal infusions; spices; fodder; products of animal origin; and derivatives (e.g., juices, fermented beverages, milk, and dairy products) are at high risk of contamination, considering their resistance to several processes used in food preparation [5]. The aflatoxins and ochratoxins (especially AFB1 and OTA) are among the most toxic and studied fungal compounds and have a significant economic and biological impact. The consumption of contaminated food and feed induces neurotoxic, immunosuppressive, and carcinogenic effects in humans and animals [6].

The fungi *Aspergillus parasiticus* and *A. flavus* are the leading producers of AFB1, which affect oilseeds, cereals, spices, milk, tree nuts, and dried fruits. In humans, AFB1 can cause mutagenicity, hepatotoxicity, immunotoxicity, nephrotoxicity, and neurotoxicity [7]. Different fungal species produce OTA, e.g., *A. westerdijkiae, A. steynii, A. niger*, *A. ochraceus*, and *Penicillium verrucosum*. OTA contaminates maize, rice, wheat, barley, sorghum, oats, wine, coffee, tea, dried fruits, vegetables, spices, milk, and cheese. The presence of OTA in food is associated with hepatotoxicity, nephrotoxicity, genotoxicity, embryotoxicity, immunotoxicity, and neurotoxicity [8].

The mycotoxin contamination can be reduced by preventing fungal proliferation on the raw material by adopting agricultural practices (i.e., appropriate irrigation and fertilization techniques for crops, pesticides, and resistant cultivars) and optimizing the postharvest transport and storage conditions by physical, chemical, and biological strategies [9,10]. The physical methods include high-pressure cooking, extrusion process, and gamma rays irradiating [11]. The chemical strategies use organic solvents, adsorbent materials (e.g., clays, alumina, silica, zeolites, aluminosilicates, and activated carbons), and essential oils [12,13]. Finally, the biocontrol approaches involve microorganisms (i.e., fungi, bacteria, and yeasts) and enzymes [14,15]. Biocontrol is an eco-friendly strategy, since it does not produce unwanted by-products and toxic residues. Many scientific papers report that fungi (i.e., species of *Aspergillus*, *Rhizopus*, and *Penicillium*) are active in reducing mycotoxins contamination [9]. Among these, the fungi of the genus *Trichoderma* are attractive for their extensive use in agriculture [16,17]. They are free-living, filamentous fungi, mostly soil-resident, including rhizosphere-competent strains [18]. *Trichoderma* fungi suppress foliar and soil-borne plant pathogens; enhance plant resistance to diseases and abiotic stresses; and promote mineral nutrition, plant growth, and crop productivity [19,20]. These beneficial fungi are competitors in the soil environment to pathogen species and synthesize secondary metabolites to decrease plant pathogens [21] by an interference competition [22]. Moreover, *Trichoderma* fungi can produce peroxidases, glucanases, chitinases, and cellulases, enzymes active against phytopathogens [23,24] used in the postharvest disease management of papaya, apple, tomato, pear, mango, banana, potato, and berries [25,26,27,28,29,30,31,32,33,34,35,36,37].

In this work, we evaluated the ability of *Trichoderma afroharzianum* strain T22 to synthesize peroxidases using different substrates. Then, we tested the effect of the exoenzymes on the degradation of mycotoxins AFB1 and OTA in different doses and at different incubation times.

## 2. Results and Discussion

### 2.1. Peroxidases Production

Mycotoxins are natural compounds produced by filamentous fungi, which, under suitable temperature and humidity conditions, may develop on various foods and feeds, causing severe risks to human and animal health [38]. Some works have been performed to safeguard foodstuffs from the contamination of mycotoxins and detoxify contaminated products [39].

**Figure 1:**
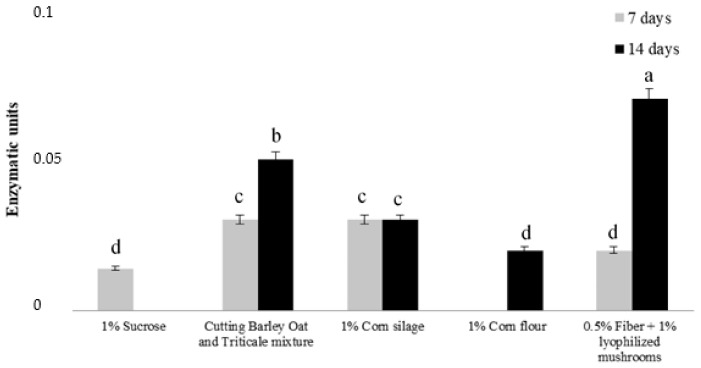
Peroxidase activity of culture filtrates produced by *Trichoderma afroharzianum* T22 at 7 and 14 days in a salt medium (SM 1X) amended with different carbon sources. Different letters indicate statistically significant differences (*p* < 0.05). Error bars represent the standard deviation.

Biological detoxification, or the enzymatic degradation of mycotoxins by microorganisms, represents an attractive approach to conventional mycotoxin inactivation. For example, *Trichoderma* fungi can synthesize several enzymes, such as peroxidase, chitinase, β-1, 3-glucanase, and phenylalanine ammonia-lyase, to overcome environmental stresses [40,41,42]. Previous works have shown the involvement of peroxidases in mycotoxin (especially OTA and AFB1) degradation and decontamination [43,44,45] as they are capable of oxidizing aflatoxins into polar and less toxic molecules [46]. The fungal strain and growing condition (i.e., media) affect the Trichoderma’s ability to produce peroxidases [47]. In this work, the ability of *T. afroharzianum* T22 to release peroxidases was evaluated on different substrates at two different times (7 and 14 days). The best performance was obtained using a medium containing lyophilized mushrooms and crude fiber (enzymatic units = 0.075) after 14 days (Figure 1). Therefore, this substrate was used to test *Trichoderma’s* ability to induce mycotoxin degradation.

### 2.2. Aflatoxin B1 and OTA In Vitro Degradation

**Figure 2:**
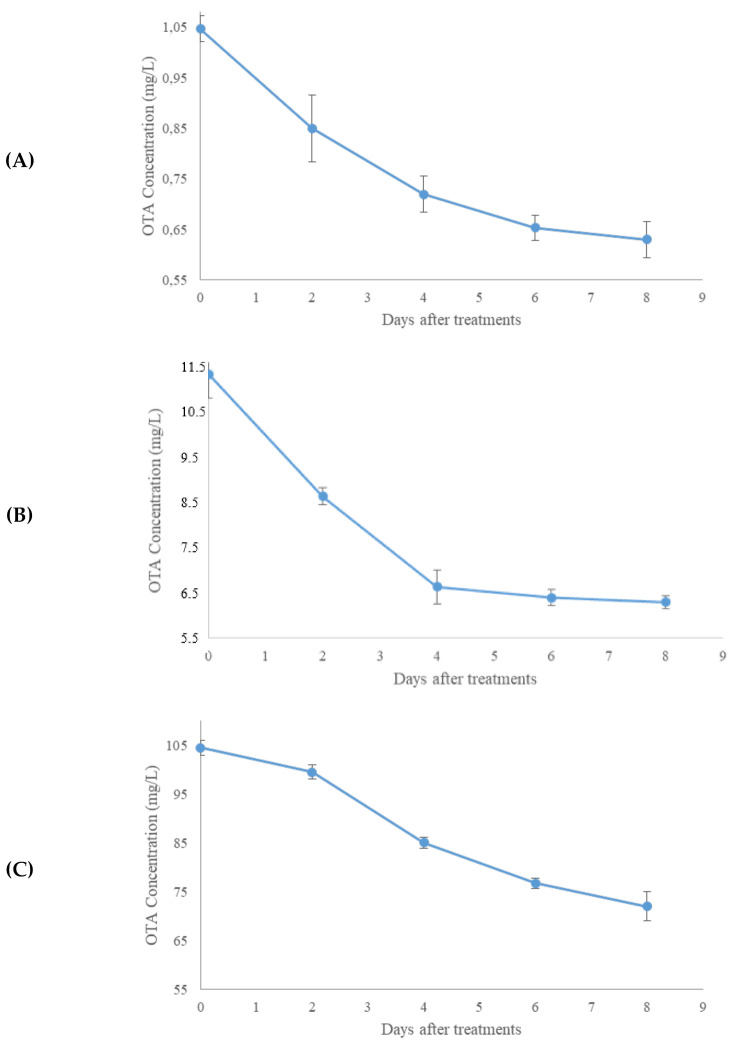
Degradation kinetics of Ochratoxin A (OTA) at different concentrations (0.001 mg/L (**A**), 0.01 mg/L (**B**), and 0.1 mg/L (**C**)) after treatment with *T. afroharzianum* strain T22 culture filtrates. Error bars represent the standard deviation, and the significant difference was set to *p* < 0.05.

The degradation of AFB1 and OTA with the T22 enzymatic mixture was determined at different incubation times, considering the importance of this parameter and pH for the enzyme activity [48]. Times and mycotoxin concentrations were chosen based on the results already reported in the literature [49,50,51]. Figure 2 reports the kinetics degradation of OTA (at 0.01, 0.1, and 1 mg/L) following treatment with *Trichoderma* enzyme mixtures. After 8 days, the concentration of OTA decreased in a different and nonproportional way from the initial mycotoxin concentration. The best degradation of OTA was achieved at 0.01 mg/L with a degradation rate of 46% (Figure 2B), followed by 37% when OTA was 0.001 mg/L (Figure 2A) and 31% when OTA was 0.1 mg/L (Figure 2C).

**Figure 3:**
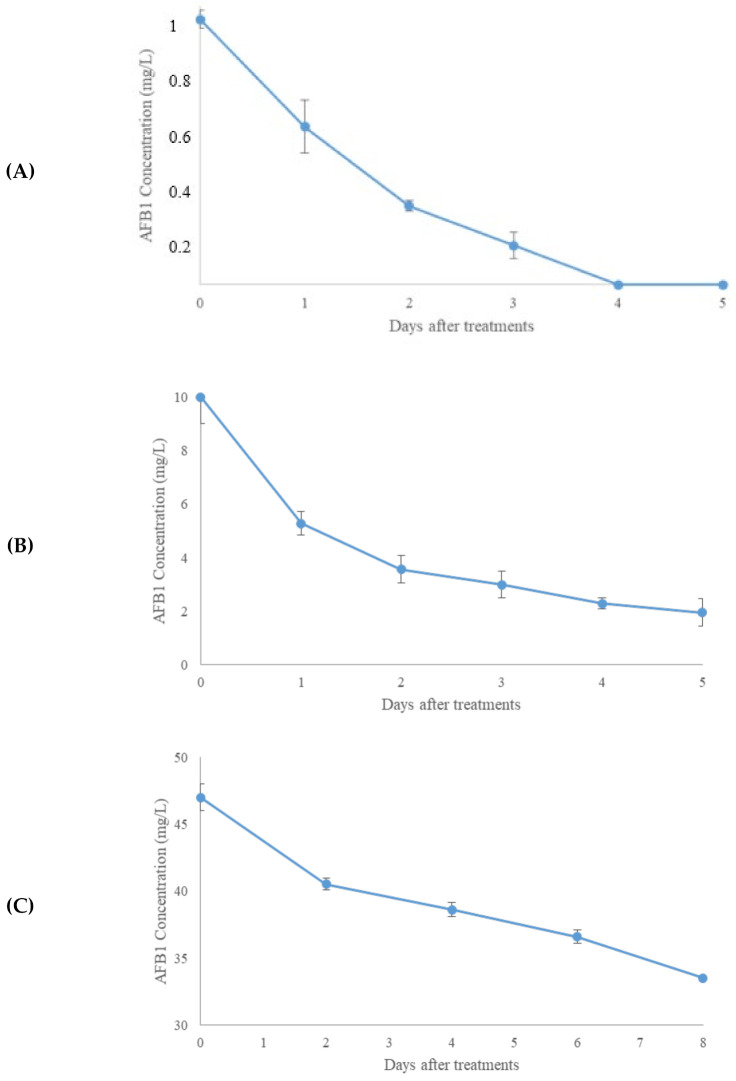
Kinetics degradation of Aflatoxin B1 (AFB1) at different concentrations (0.001 mg/L (**A**), 0.01 mg/L (**B**), and 0.1 mg/L (**C**)) after treatment with *T. afroharzianum* strain T22 culture filtrates. Error bars represent the standard deviation, and the significant difference was *p* < 0.05.

Figure 3A–C reports the kinetics degradation of AFB1. After 8 days of incubation, the degradation rate was 100% at 0.001 mg/L, 63% at 0.01 mg/L, and 28% at 0.1 mg/L. Thus, the degradation of AFB1 after treatment with the T22 culture filtrate was inversely proportional to the mycotoxin concentration.

The reduction of both mycotoxins confirmed the involvement of fungal enzymes in the mycotoxin degradation (Figure 2 and Figure 3). In both cases, the optimum reduction time was 8 days. The different decontamination yields depending on the dose of mycotoxin treated suggested a relationship between the levels of decontaminating agent and toxin. Different mechanisms could explain the slightly different behaviors of the two enzymatic kinetics. The T22 culture filtrate decontamination inversely proportional to the mycotoxin concentration could depend on the direct action of peroxidases on AFB1, which generates lower toxicity products (i.e., Aflatoxins P1, M1, B2a, Q1, and aflatoxicol) by opening the difurocoumarin moiety of Aflatoxin B1 and hydrolyzing the vinylene group [52]. The changes in the lactone and furan rings negatively affect the AFB1 toxicity, since the unsaturated 8,9-bond in the furofuran ring and the lactone ring are interested in the AFB1–DNA and AFB1–protein interactions [50,51]. The severity’s order of toxicity (acute to chronic: AFB1 > AFB2 > AFM1) is ascribable to the chirality, steric hindrances, and resonance energy [53]. It has not fully elucidated the peroxidase action on ochratoxin A. It is known that the OTA contamination determines ROSs production, lipid peroxidation, DNA damage, and the loss of cell function [54]. ROSs production is due to the inhibition of antioxidative enzymes. Lipid peroxidation determines an increase in the malondialdehyde (MDA) level [54].

On the contrary, *Trichoderma* spp. releases antioxidant enzymes and decreases the MDA content [54]. Therefore, at the same concentration of *Trichoderma* culture filtrates and different OTA levels, the peroxidase level decreases as the OTA concentration increases. Moreover, the maximum degradation levels of OTA occurred at 0.01 mg/L and could depend on the release of H_2_O_2_ in the reaction mixture. H_2_O_2_ is the peroxidase cosubstrate, which can create an intermediate molecule that transforms a phenolic substrate into a free radical [54]. It is known that the peroxidases reveal a kinetic performance of inactivation, both in the excess or absence of H_2_O_2_ [55]. Noteworthy, the prolongation of detoxification for one week at 37 °C suggests that these enzymes are stable at this time and temperature.

### 2.3. Aflatoxin B1 and OTA Degradation on Maize Flour and Enzyme Isolation

**Figure 4:**
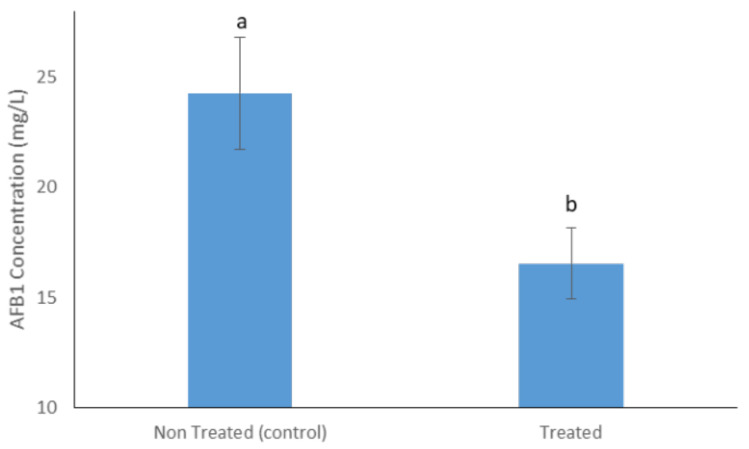
Aflatoxin B1 concentration in contaminated maize flour (AFB1 at 0.1 mg/L) treated with the enzymatic mixture produced by *T. afroharzianum* strain T22 (treated) and non-treated (control). Different letters indicate statistically significant differences (*p* < 0.05). Error bars represent the standard deviation.

The formulation based on T22 exoenzymes, tested on maize flour artificially contaminated with Aflatoxin B1 (0.1 mg/L), decreased (−30%) the AFB1 levels (Figure 4).

Numerous evidence supports the application of peroxidases as food additives to degrade mycotoxins in food and feed [56]. They are easier to dose and store than producing fungi while retaining the ability to make nontoxic or less toxic products than chemical approaches.

**Figure 5:**
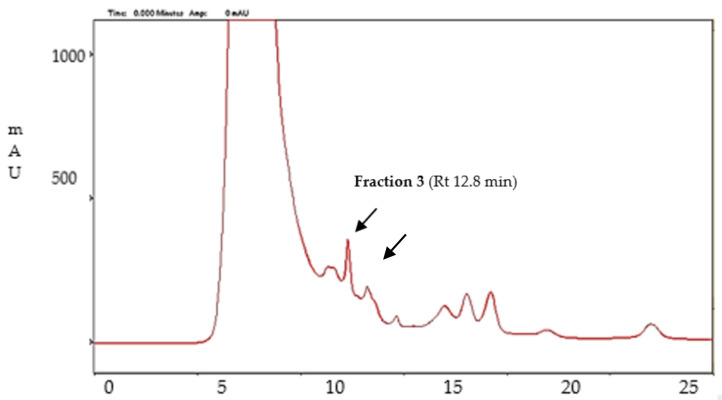
Chromatogram of *T. afroharzianum* T22 culture filtrates obtained by HPLC separation. Arrow indicates the fraction (number 3, Rt 12.8 min) with the best degradation activity.

HPLC gel filtration chromatography and SDS-page electrophoresis were used to isolate the enzyme responsible for the mycotoxin degradation. The HPLC chromatogram of *T. afroharzianum* strain T22 culture filtrates (Figure 5) showed ten main peaks. Standard solutions of AFB1 were added to each isolated fraction to test the degrading activity. Fraction number 3 (peak at Rt 12.8 min—Figure 5) showed the best activity, with a degradation rate of 100% at 0.001 mg/L, 52% at 0.01 mg/L, and 51.63% at 0.1 mg/L (data not shown). SDS page electrophoresis showed three prominent bands that were isolated and used to determine which was responsible for the AFB1 reduction. Fraction 2 gave the maximum degradation percentage of AFB1. Thus, it was possible to assume that one or more *Trichoderma* enzymes with a molecular weight range of 60–90 kDa were involved in the degradation of this mycotoxin.

## 3. Material and Methods

### 3.1. Fungal Strains and Growing Conditions

A pure culture of the *Trichoderma afroharzianum* strain T22 was obtained from the collection available at the Department of Agricultural Sciences of the University of Naples Federico II, Italy. Fungal cultures were incubated at 25 °C on Petri dishes containing Potato Dextrose Agar (PDA; HiMedia Laboratories, Mumbai, India). After 7–10 days, the spores were collected in a 20% (*v*/*v*) glycerol solution, and the concentration of conidial suspensions was determined using a counting chamber.

Conidia suspensions (1 × 10^8^ spores/mL; 100 μL) were inoculated in 250-mL flasks containing 100 mL of Potato Dextrose Broth (PDB; Carlo Erba, Milano, Italy) and incubated under shaking for three days at 25 °C. The biomass was filtered (Miracloth; Calbiochem; Merck, Darmstadt, Germany) and transferred aseptically into flasks (1-L) containing 500 mL of salt medium (SM) at pH 6.6 [57]. The SM contained KH_2_PO_4_ 680 mg L^−1^, KCl 200 mg L^−1^, K_2_HPO_4_ 870 mg L^−1^, CaCl_2_ 200 mg L^−1^, NH_4_NO_3_ 1 g L^−1^, FeSO_4_ 2 mg L^−1^, MgSO_4_∙7H_2_O 200 mg L^−1^, MnSO_4_ 2 mg L^−1^, sucrose 10 g L^−1^, ZnSO_4_ 2 mg L^−1^, and agar 10 g L^−1^. The SM was amended with: sucrose (1%, *w*/*v*), a barley oat and triticale mixture (1%, *w*/*v*), corn silage (1%, *w*/*v)*, cornflour (1%, *w*/*v*), or wheat fiber (0.5% *w*/*v*) and lyophilized mushrooms (*Agaricus bisporus* basidiocarps; 1%, *w*/*v*).

The chemicals were purchased by Merck (Darmstadt, Germany).

The cultures were incubated starting from 7 up to 14 days under continuous agitation (120 rpm, at 25 °C), then centrifuged at 4000 rpm for 20 min. The supernatants were filter-sterilized using three different Millipore filters (pores of 0.8, 0.45, and 0.22-μm diameters; Merck) in decreasing succession, and the filtrates were dialyzed against distilled water (48 h at 4 °C) using membranes with 6–8-kDa pore sizes.

### 3.2. Peroxidase Assay

The assay is based on the reaction between hydrogen peroxide, phenol, and aminoantipyrine catalyzed by peroxidase, which produces a pink color whose intensity is proportional to the concentration of the enzyme.

The culture filtrate (50 μL) was added to hydrogen peroxide 0.0017 M (1.5 mL; pH 7.1) (Thermo Fisher Scientific, Waltham, MA, USA), 4-aminoantipyrine solution 0.0025 M (4 mL; Thermo Fisher Scientific), and phenol 0.17 M (Thermo Fisher Scientific). Distilled water was added to the reagents and used as a blank.

The absorbance was read at 510 nm (time = 5 min) in a spectrophotometer (Helios β, Unicam; Thermo Fisher Scientific).

The enzymatic activity was calculated as the difference between the final and initial absorbances. The values obtained were transformed into enzymatic units by comparison with the calibration line (Figure S1) made using increasing concentrations of commercial “horseradish peroxidase” (Merck).

### 3.3. Degradation of Aflatoxin B1 and Ochratoxin A Using Enzymatic Mixtures Produced by Trichoderma

The enzymatic mixture produced by *T. afroharzianum* strain T22 was concentrated 20 times and dialyzed against distilled water (100 μL). Subsequently, 800 μL of physiological solution (containing 0.9% sodium chloride in water, Merck) and 100 μL of AFB1 (Sigma-Aldrich, Merck, Darmstadt, Germany) or OTA (Sigma-Aldrich) at concentrations of 0.01, 0.1, and 1 mg/L were added. The tubes were incubated under agitation for 8 days at 37 °C. Every 2 days, a sample (20 μL) was analyzed by High-Performance Liquid Chromatography (HPLC), as described below.

#### 3.3.1. Aflatoxin B1 Quantification

The quantification of AFB1 in liquid samples and planta matrices was obtained by an HPLC method [47]. The chromatographic separations were carried out in a Shimadzu HPLC system (Shimadzu, Kyoto, Japan) equipped with LC-10 ADBP pumps, a SCL 10 ABP controller fluorimetric detector (λ 360 nm absorption, λ 440 nm emission), a C18 column with a 5-mm particle diameter (Phenomenex Gemini, Torrance, CA, USA), and a security Guard C-18 pre-column. The mobile phase was water:acetonitrile:methanol (50:25:25, *v*/*v*) (Sigma-Aldrich). The flow rate was 1 mL/min. The retention time of AFB1 was 8.5 min.

The analyses were performed in triplicate, and the results were analyzed with Class Vp software (Shimadzu). The areas underlying the peaks were transformed into mg/L by comparison with a calibration line obtained with different concentrations of the AFB1 standard solutions. Figure S1 reported the AFB1 calibration line.

#### 3.3.2. Ochratoxin A Quantification

The quantification of OTA was obtained by the HPLC method [46]. The chromatographic separations were carried out in the isocratic mode using 65% phase A (water + 1% acetic acid) and 35% phase B (acetonitrile + 1% acetic acid) (Sigma-Aldrich). The flow rate was 1 mL/min. The injection volume was 20 μL. The experiments were performed using a Shimadzu HPLC system (Shimadzu), equipped as described above, and a Bio-Sil C18 HL 90-5 column with a diameter of 5-mm particles (4.6 × 250 mm, Bio-Rad, Richmond, CA, USA). The retention time of OTA was 5.5 min. The data were processed using Class Vp software (Shimadzu). The areas underlying the peaks were transformed into mg/L by comparison with a calibration line obtained with different concentrations of OTA standard solutions. Figure S2 reported the OTA calibration line.

### 3.4. Degradation of Aflatoxin B1 in Cornflour

The *Trichoderma* T22 enzymatic mixture’s ability to degrade AFB1 was evaluated when inoculated on cornflour (solid matrix) with a known concentration of AFB1 (Sigma). Contamination was carried out on maize flour samples obtained by grinding 300 g of kernels. The flour was autoclaved (120 °C for 5 min) and cooled with a laminar flow hood for 6 h. Aliquots of 50 g were placed in Petri dishes (diameter = 20 cm; Thermo Fisher Scientific) containing 15 mL of AFB1 (1 mg/L). The solvent was removed by evaporation under a fume hood at room temperature. Triplicate samples were treated with the enzymatic mixture (5 mL), dialyzed, and then concentrated (20 times). Three samples treated with 25 mL of sterile distilled water were used as controls. The extraction of AFB1 was carried out after 8 days of incubation (37 °C), according to the Vicam Afla test method (VICAM, Milford, MA, USA), and the quantification was performed by HPLC analysis, as described previously.

### 3.5. Extraction of Aflatoxin B1 from Cornflour

The extraction of AFB1 from cornflour was carried out according to Vicam’s Afatest instruction manual (VICAM).

Briefly, the samples (50 g) were homogenized in Ultraturrax T25 Basic (IKA Werke, Staufen, Germany) with sodium chloride (5 g) (Sigma-Aldrich) and were added to an 80:20 (*v*/*v*) methanol/water solution (100 mL; Sigma-Aldrich). Then, the samples were filtered on Whatman no. 4 filter paper (Whatman, Maidstone, UK), diluted with distilled water (40 mL), and filter-sterilized on 0.20-μm discs (Whatman). Each sample (10 mL) was chromatographed on IAC Afla Test (Vicam) immunoaffinity columns at 1 drop/second. The column was washed with water (10 mL). The methanol (1 mL, Sigma-Aldrich) was used to elute AFB1.

The eluate (20 μL) was used to evaluate the degradation levels of AFB1 by HPLC analysis, as previously described.

### 3.6. Fractionation of Enzymatic Mixtures Involved in Aflatoxin B1 Degradation

#### 3.6.1. Gel Filtration

The culture filtrate of *Trichoderma* T22, used for the AFB1 degradation assays, was fractionated by HPLC (Shimadzu), equipped as described above, using a gel filtration Superdex 75 HR 10/30 column (Pharmacia LKB Biotechnology, Uppsala, Sweden). The chromatographic course was conducted under isocratic conditions (1 mL/min flow) using water as a mobile phase.

#### 3.6.2. Electrophoresis

The fractions were concentrated to obtain a final volume of 1 mL. The samples (4 μL) were diluted in the running buffer (5 μL of β-mercaptoethanol and a 95-μL Laemmli sample buffer) (Pharmacia LKB Biotechnology) and separated by vertical electrophoresis under tricine denaturing conditions -SDS-PAGE (PHAST GEL^®^ electrophoresis system, Pharmacia LKB Biotechnology).

The running gel contained 12% polyacrylamide; the separation gel contained 4% polyacrylamide in Tris 3 M buffer + 0.3% SDS.

The running buffers were made with 0.1 M Tris, 0.1 M tricine, and 0.1% SDS pH 8.5 at the cathode and 0.2 M Tris-HCl pH 8.9 at the anode.

The time course was 30 min, the power 80 volts. The gel at the end of the electrophoretic course was colored with silver nitrate according to the procedure described by Blum et al. [36]. The gel was fixed (10 min) in a solution containing 50% ethanol and 10% acetic acid (*v*/*v*) to remove the electrophoresis buffer and the urea. Then, it was washed (three minutes) with deionized water, incubated (1 min) in a solution consisting of 0.02 g/100 mL of sodium thiosulfate pentahydrate, and washed with deionized water (3 times for 30 s). After fixing, the gel was placed (20 min) in a staining solution (containing 0.1 g/50 mL of silver nitrate + 38 μL of 37% formaldehyde). Subsequently, it was rinsed in deionized water (twice for 20 s) to remove the silver in excess and developed in an alkaline solution containing sodium carbonate (0.3 g/50 mL) + 37% formaldehyde (25 μL) + 1 mL Na_2_S_2_O_3_∙5H_2_O (0.02 g solution/100 mL). When visible bands appeared on the gel, two rinses of 2 min were carried out to eliminate the excess reagents, and the reaction was stopped with a solution containing ethanol (50%) + acetic acid (10%). The gel was analyzed using Imaging Densitometer GS-800 (Bio-Rad), which allowed the acquisition of a digital image and subsequent analysis with Quantity One software (Bio-Rad).

All chemicals were purchased by Honeywell Fluka (Charlotte, NC, USA).

### 3.7. Statistical Analysis

Statistical analyses were made using Statistica software (StatSoft, Tulsa, OK, USA). The differences among the means were tested at *p* < 0.05.

## 4. Conclusions

Contamination of agricultural products with mycotoxins is dangerous for animal and human health. The risk of mycotoxicosis can be reduced by using beneficial microorganisms and their enzymes. Numerous studies have demonstrated the ability of *Trichoderma* spp. as a biocontrol agent, but only a few studies have been concerned about its ability to degrade mycotoxins. For the first time, our results demonstrated the ability of *Trichoderma afroharzianum* T22 to degrade AFB1 and OTA using an inducing media to release exohydrolases with peroxidase activity. High levels of degradation have been demonstrated both in vitro (up to 100% of degradation at a low concentration of AFB1) and in contaminated maize flour (30% of degradation of AFB1).

The results obtained in this study showed that T22 or *Trichoderma* peroxidase supplementation could represent sustainable strategies for the degradation of AFB1 and OTA. The characterization of *Trichoderma* peroxidases, the adverse effects of enzyme preparations on food/feed, and the toxicity of the degradation products of the selected mycotoxins are underway in our laboratory.

## Data Availability

The data is contained within the article or Supplementary Materials.

## Supplementary Materials

The following are available online at https://www.mdpi.com/article/10.3390/molecules27123959/s1: Figure S1: Aflatoxin B1 calibration line and Figure S2: Ochratoxin A calibration line.

## References

[1] Agriopoulou S., Stamatelopoulou E., Varzakas T. (2020). Advances in Analysis and Detection of Major Mycotoxins in Foods. Foods.

[2] Grenier B., Applegate T.J. (2013). Modulation of Intestinal Functions Following Mycotoxin Ingestion: Meta-Analysis of Published Experiments in Animals. Toxins.

[3] Udovicki B., Audenaert K., De Saeger S., Rajkovic A. (2018). Overview on the mycotoxins incidence in Serbia in the period 2004–2016. Toxins.

[4] Ostry V., Malir F., Toman J., Grosse Y. (2017). Mycotoxins as human carcinogens-the IARC Monographs classification. Mycotoxin Res..

[5] Fanelli F., Cozzi G., Raiola A., Dini I., Mulè G., Logrieco A.F., Ritieni A. (2017). Raisins and Currants as Conventional Nutraceuticals in Italian Market: Natural Occurrence of Ochratoxin A. Food Sci. J..

[6] Enyiukwu D.N., Awurum A.N., Nwaneri J.A. (2014). Mycotoxins in stored agricultural products: Implications to food safety and health and prospects of plant-derived pesticides as novel approach to their management. Greener J. Microbiol. Antimicrob..

[7] Frangiamone M., Cimbalo A., Alonso-Garrido M., Vila-Donat P., Manyes L. (2021). In vitro and in vivo evaluation of AFB1 and OTA-toxicity through immunofluorescence and flow cytometry techniques: A systematic review. Food Chem. Toxicol..

[8] Frangiamone M., Alonso-Garrido M., Font G., Cimbalo A., Manyes L. (2022). Pumpkin extract and fermented whey individually and in combination alleviated AFB1-and OTA-induced alterations on neuronal differentiation in vitro. Food Chem. Toxicol..

[9] Čolović R., Puvača N., Cheli F., Avantaggiato G., Greco D., Đuragić O., Kos J., Pinotti L. (2019). Decontamination of Mycotoxin-Contaminated Feedstuffs and Compound Feed. Toxins.

[10] Çelik K., Rai M., Abd-Elsalam K.A. (2020). The efficacy of mycotoxin-detoxifying and biotransforming agents in animal nutrition. Nanomycotoxicology.

[11] Conte G., Fontanelli M., Galli F., Cotrozzi L., Pagni L., Pellegrini E. (2020). Mycotoxins in Feed and Food and the Role of Ozone in Their Detoxification and Degradation: An Update. Toxins.

[12] Haque M.D., Wang Y., Shen Z., Li X., Saleemi M.K., He C. (2020). Mycotoxin contamination and control strategy in human, domestic animal and poultry: A review. Microb. Pathog..

[13] Dini I. (2016). Chapter 14—Use of Essential Oils in Food Packaging. Essential Oils in Food Preservation, Flavor and Safety.

[14] Moncini L., Sarrocco S., Pachetti G., Moretti A., Haidukowski M., Vannacci G. (2020). N_2_ controlled atmosphere reduces postharvest mycotoxin risk and pests attack on cereal grains. Phytoparasitica.

[15] Alberts J.F., Lilly M., Rheeder J.P., Burger H.M., Shephard G.S., Gelderblom W.C.A. (2017). Technological and community-based methods to reduce mycotoxin exposure. Food Control.

[16] Dini I., Marra R., Cavallo P., Pironti A., Sepe I., Troisi J., Scala G., Lombari P., Vinale F. (2021). *Trichoderma* Strains and Metabolites Selectively Increase the Production of Volatile Organic Compounds (VOCs) in Olive Trees. Metabolites.

[17] Vinale F., Marra R., Scala F., Ghisalberti E., Lorito M., Sivasithamparam K. (2006). Major secondary metabolites produced by two commercial *Trichoderma* strains active against different phytopathogens. Lett. Appl. Microbiol..

[18] Harman G.E., Howell C.R., Viterbo A., Chet I., Lorito M. (2004). *Trichoderma* species—Opportunistic, avirulent plant symbionts. Nat. Rev. Microbiol..

[19] Altomare C., Norvell W.A., Björkman T., Harman G.E. (1999). Solubilization of Phosphates and Micronutrients by the Plant-Growth Promoting and Biocontrol Fungus *Trichoderma harzianum* Rifai 1295-22. Appl. Environ. Microbiol..

[20] López-Bucio J., Pelagio-Flores R., Herrera-Estrella A. (2015). *Trichoderma* as biostimulant: Exploiting the multilevel properties of a plant beneficial fungus. Sci. Hortic..

[21] Hermosa R., Cardoza R.E., Rubio M.B., Gutiérrez S., Monte E., Gupta V.K., Schmoll M. (2014). Secondary metabolism and antimicrobial metabolites of *Trichoderma*. Biotechnology and Biology of Trichoderma.

[22] Ren X., Branà M.T., Haidukowski M., Gallo A., Zhang Q., Logrieco A.F., Li P., Zhao S., Altomare C. (2022). Potential of *Trichoderma* spp. for Biocontrol of Aflatoxin-Producing *Aspergillus flavus*. Toxins.

[23] Vinale F., Sivasithamparam K., Ghisalberti E.L., Marra R., Woo S.L., Lorito M. (2008). *Trichoderma*-plant-pathogen interactions. Soil Biol. Biochem..

[24] Valenzuela N.L., Angel D.N., Ortiz D.T., Rosas R.A., García C.F.O., Santos M.O. (2015). Biological Control of Anthracnose by Postharvest Application of *Trichoderma* spp. on Maradol Papaya Fruit. Biol. Control.

[25] Dal Bello G., Lampugnani G., Abramoff C., Fusé C., Perelló A. (2015). Postharvest Control of Botrytis Gray Mould in Tomato by Antagonists and Biorational Compounds. IOBC-WPRS Bull..

[26] Quaglia M., Ederli L., Pasqualini S., Zazzerini A. (2011). Biological Control Agents and Chemical Inducers of Resistance for Postharvest Control of *Penicillium expansum* Link. on Apple Fruit. Postharvest Biol. Technol..

[27] Batta Y.A. (2004). Effect of Treatment with *Trichoderma harzianum* Rifai Formulated in Invert Emulsion on Postharvest Decay of Apple Blue Mold. Int. J. Food Microbiol..

[28] Mortuza M.G., Ilag L.L. (1999). Potential for Biocontrol of *Lasiodiplodia theobromae* (Pat.) Griff. & Maubl. in Banana Fruits by *Trichoderma* Species. Biol. Control.

[29] Sangeetha G., Usharani S., Muthukumar A. (2009). Biocontrol with *Trichoderma* Species for the Management of Postharvest Crown Rot of Banana. Phytopathol. Mediterr..

[30] Dania V.O. (2019). Bioefficacy of *Trichoderma* Species against Important Fungal Pathogens Causing Post-Harvest Rot in Sweet Potato (*Ipomoea batatas* (L.) Lam). J. Bangladesh Agric. Univ..

[31] Prabakar K., Raguchander T., Saravanakumar D., Muthulakshmi P., Parthiban V.K., Prakasam V. (2008). Management of Postharvest Disease of Mango Anthracnose Incited by *Colletotrichum gleosporioides*. Arch. Phytopathol. Plant Prot..

[32] Nallathambi P., Umamaheswari C., Thakore B.B.L., More T.A. (2009). Post-Harvest Management of Ber (*Ziziphus mauritiana* Lamk) Fruit Rot (*Alternaria alternata* Fr. Keissler) Using *Trichoderma* Species, Fungicides and Their Combinations. Crop Prot..

[33] Harman G.E., Hayes C.K., Lorito M., Broadway R.M., Di Pietro A., Tronsmo A. (1993). Chitinolytic enzymes of *Trichoderma harzianum*: Purification of chitobiosidase and endochitinase. Phytopathology.

[34] Jalili M., Jinap S., Adzahan N. (2009). Survey of aflatoxins in retail samples of whole and ground black and white peppercorns. Food Addit. Contam..

[35] Jalili M., Jinap S., Radu S. (2010). Natural occurrence of ochratoxin A contamination in commercial black and white pepper products. Mycopathologia.

[36] Blum H., Beier H., Gross H.J. (1987). Improved silver staining of plant proteins, RNA and DNA in polyacrylamide gels. Electrophoresis.

[37] Alshannaq A., Yu J.H. (2017). Occurrence, Toxicity, and Analysis of Major Mycotoxins in Food. Int. J. Environ. Res. Public Health.

[38] Shcherbakova L.A. (2019). Fungicide resistance of plant pathogenic fungi and their chemosensitization as a tool to increase anti-disease effects of triazoles and strobilurines. Sel’skokhozyaistvennaya Biol..

[39] Saravanakumar K., Li Y., Yu C., Wang Q.Q., Wang M., Sun J., Chen J. (2017). Effect of *Trichoderma harzianum* on maize rhizosphere microbiome and biocontrol of *Fusarium* stalk rot. Sci. Rep..

[40] Eslahi N., Kowsari M., Zamani M., Motallebi M. (2021). The profile change of defense pathways in *Phaseouls vulgaris* L. by biochemical and molecular interactions of *Trichoderma harzianum* transformants overexpressing a chimeric chitinase. Biol. Control.

[41] Iannaccone F., Alborino V., Dini I., Balestrieri A., Marra R., Davino R., Di Francia A., Masucci F., Serrapica F., Vinale F. (2022). In Vitro Application of Exogenous Fibrolytic Enzymes from *Trichoderma* spp. to Improve Feed Utilization by Ruminants. Agriculture.

[42] Ijoma G.N., Selvarajan R., Tekere M. (2019). The potential of fungal co-cultures as biological inducers for increased ligninolytic enzymes on agricultural residues. Int. J. Environ. Sci. Technol..

[43] Nora N.S., Feltrin A.C.P., Sibaja K.V.M., Furlog E.B., Garda-Buffon J. (2019). Ochratoxin A reduction by peroxidase in a model system and grape juice. Braz. J. Microbiol..

[44] Tripathi S., Mishra H.N. (2011). Modeling and optimization of enzymatic degradation of aflatoxin B_1_ (AFB_1_) in red chili powder using response surface methodology. Food Bioprocess Technol..

[45] Hackbart H.C.S., Machado A.R., Christ-Ribeiro A., Prietto L., Badiale-Furlong E. (2014). Reduction of aflatoxins by *Rhizopus oryzae* and *Trichoderma reesei*. Mycotoxin Res..

[46] De Oliveira F.K., Santos L.O., Buffon J.G. (2021). Mechanism of action, sources, and application of peroxidases. Food Res. Int..

[47] Motamed S., Ghaemmaghami F., Alemzadeh I. (2009). Turnip (*Brassica rapa*) peroxidase: Purification and characterization. Ind. Eng. Chem. Res..

[48] Alberts J.F., Engelbrecht Y., Steyn P.S., Holzapfel W.H., Van Zyl W.H. (2006). Biological degradation of aflatoxin B1 by *Rhodococcus erythropolis* cultures. Int. J. Food Microbiol..

[49] Teniola O.D., Addo P.A., Brost I.M., Farber P., Jany K.D., Alberts J.F., Van Zyl W.H., Steyn P.S., Holzapfel W.H. (2005). Degradation of aflatoxin B1 by cell-free extracts of *Rhodococcus erythropolis* and *Mycobacterium fluoranthenivorans* sp. nov. DSM44556. Int. J. Food Microbiol..

[50] Cheng-An H., Draughan F.A. (1994). Degradation of Ochratoxin A by *Acinetobacter calcoaeticus*. J. Food Prot..

[51] Braun H., Woitsch L., Hetzer B., Geisen R., Zange B., Schmidt-Heydt M. (2018). *Trichoderma harzianum*: Inhibition of mycotoxin producing fungi and toxin biosynthesis. Int. J. Food Microbiol..

[52] Denardi de Souza T., Caldas S.S., Primel E.G., Furlong E.B. (2015). Exposure to deoxynivalenol, Ht-2 and T-2 toxins by consumption of wheat-based product in southern Brazil. Food Control.

[53] Guan Y., Chen J., Nepovimova E., Long M., Wu W., Kuca K. (2021). Aflatoxin Detoxification Using Microorganisms and Enzymes. Toxins.

[54] Adebo O., Njobeh P., Gbashi S., Nwinyi O., Mavumengwana V. (2017). Review on microbial degradation of aflatoxins. Crit. Rev. Food Sci. Nutr..

[55] Magouz F., Abu-Ghanima H., Zaineldin A.I., Gewaily M.S., Soliman A., Amer A.A., Moustafa E.M., Younis E.M., Abdel-Warith A.-W.A., Davies S.J. (2022). Dietary *Bacillus subtilis* relieved the growth retardation, hepatic failure, and antioxidative depression induced by ochratoxin A in Thinlip Mullet (*Liza ramada*). Aquac. Rep..

[56] Mei L., Guang–Shu M.A., Lian H., Su X., Tian Y., Huang W., Mei J., Jiang X. (2019). The effects of *Trichoderma* on preventing cucumber fusarium wilt and regulating cucumber physiology. J. Integr. Agric..

[57] De Oliveira Garcia S., Sibaja K.V.M., Nogueira W.V., Feltrin A.C.P., Pinheiro D.F.A., Cerqueira M.B.R., Furlong E.B., Garda-Buffon J. (2020). Peroxidase as a simultaneous degradation agent of ochratoxin A and zearalenone applied to model solution and beer. Food Res. Int..

