# Supplementary material for: Trichoderma Enzymes for Degradation of Aflatoxin B1 and Ochratoxin A
Source: Molecules. 2022 Jun 20;27(12):3959. doi: 10.3390/molecules27123959 (PMC9231114; doi:10.3390/molecules27123959)
Supplement: Supplementary file 1 [file molecules-27-03959-s001.zip › molecules-1758549-supplementary.pdf]

molecules

MDPI

Supplementary Materials

Trichoderma Enzymes for Degradation of Aflatoxin B1 and Ochratoxin A

Irene Dini [1,*], Vittoria Alborino [2], Stefania Lanzuise [2], Nadia Lombardi [2,3], Roberta Marra [2,3], Anna Balestrieri [4], Alberto Ritieni [1], Sheridan L. Woo [1,3] and Francesco Vinale [3,5,*]

[1] Department of Pharmacy, University of Naples Federico II, Via Domenico Montesano 49, 80131 Naples, Italy; alberto.ritieni@unina.it (A.R.); sheridanlois.woo@unina.it (S.L.W.)
[2] Department of Agricultural Sciences, University of Naples Federico II, Via Università 100, 80055 Portici, Italy; agr.vittoria.alborino@gmail.com (V.A.); stefania.lanzuise@unina.it (S.L.); nadia.lombardi@unina.it (N.L.); robmarra@unina.it (R.M.)
[3] BAT Center—Interuniversity Center for Studies on Bioinspired Agro-Environmental Technology, University of Naples Federico II, Via Università 100, 80055 Portici, Italy
[4] Department of Animal Health, Istituto Zooprofilattico Sperimentale del Mezzogiorno, Via della Salute, 2 80055 Portici, Italy; anna.balestrieri@izsmportici.it
[5] Department of Veterinary Medicine and Animal Productions, University of Naples Federico II, Via Federico Delpino 1, 80138 Naples, Italy
* Correspondence: irdini@unina.it (I.D.); frvinale@unina.it (F.V.)

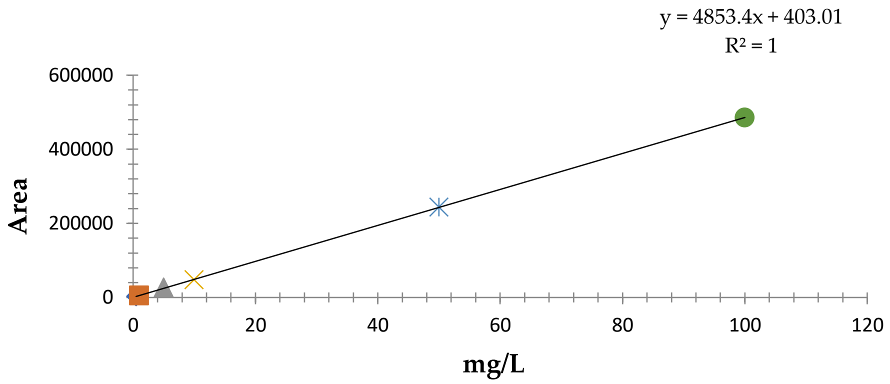

Figure S1. Aflatoxin B1 calibration line.

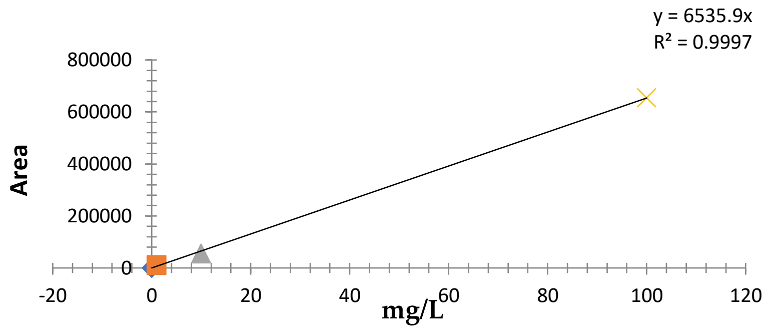

Figure S2. Ochratoxin A calibration line.
